# Leg Fidgeting Improves Executive Function following Prolonged Sitting with a Typical Western Meal: A Randomized, Controlled Cross-Over Trial

**DOI:** 10.3390/ijerph19031357

**Published:** 2022-01-26

**Authors:** Simon Fryer, Craig Paterson, Lee Stoner, Meghan A. Brown, James Faulkner, Louise A. Turner, Aitor Martínez Aguirre-Betolaza, Gabriel Zieff, Keeron Stone

**Affiliations:** 1 School of Sport and Exercise, University of Gloucestershire, Gloucestershire GL2 9HW, UK; cpaterson2@glos.ac.uk (C.P.); meghan.brown@leedsbeckett.ac.uk (M.A.B.); lturner14@glos.ac.uk (L.A.T.); kstone1@glos.ac.uk (K.S.); 2 Department of Sport and Exercise, University of North Carolina, Chapel Hill, NC 27514, USA; dr.l.stoner@gmail.com (L.S.); ghzieff@gmail.com (G.Z.); 3 Carnegie School of Sport, Leeds Beckett University, Leeds LS6 3QT, UK; 4 Department of Sport and Exercise, University of Winchester, Hampshire SO22 4NR, UK; James.Faulkner@winchester.ac.uk; 5 Department of Physical Education and Sport, Faculty of Education and Sport, University of the Basque Country (UPV/EHU), 01006 Vitoria-Gasteiz, Spain; aitor.martinezdeaguirre@ehu.eus

**Keywords:** sedentary behaviour, mental performance, cerebrovascular function, cognition, nutrition

## Abstract

Prolonged uninterrupted sitting and a typical Western meal, high in fat and refined sugar, can additively impair cognitive and cerebrovascular functions. However, it is unknown whether interrupting these behaviours, with a simple desk-based activity, can attenuate the impairment. The aim of this study was to determine whether regular leg fidgeting can off-set the detrimental effects of prolonged sitting following the consumption of a typical Western meal, on executive and cerebrovascular function. Using a randomized cross-over design, 13 healthy males consumed a Western meal and completed 180-min of prolonged sitting with leg fidgeting of 1 min on/4 min off (intervention [INT]) and without (control [CON]). Cognitive function was assessed pre and post sitting using the Trail Maker Test (TMT) parts A and B. Common carotid artery (CCA) blood flow, as an index of brain flow, was measured pre and post, and cerebral (FP1) perfusion was measured continuously. For TMT B the CON trial significantly increased (worsened) completion time (mean difference [MD] = 5.2 s, *d* = 0.38), the number of errors (MD = 3.33, *d* = 0.68) and cognitive fatigue (MD = 0.73, *d* = 0.92). Compared to CON, the INT trial significantly improved completion time (MD = 2.3 s, *d* = 0.97), and prevented declines in cognitive fatigue and a reduction in the number of errors. No significant changes in cerebral perfusion or CCA blood flow were found. Leg fidgeting for 1-min on/4-min off following a meal high in fats and refined sugars attenuated the impairment in executive function. This attenuation in executive function may not be caused by alterations in CCA blood flow or cerebral perfusion.

## 1. Introduction

Epidemiological studies have associated high amounts of sedentary behaviours with cerebrovascular and neurodegenerative diseases such as stroke, dementia, impaired brain structure and function [1], and cognitive impairment [2]. Whilst the exact mechanisms by which sedentary behaviour leads to cognitive dysfunction is unclear, it is known that periods of prolonged uninterrupted sitting can lead to reductions in brain blood flow and cerebral oxygenation which have been linked to cognitive decline [3,4], and brief physical activity interruptions can prevent these cerebrovascular declines [4,5,6] and increase memory storage [7]. In turn, habitual physical activity is associated with a lower risk of developing cerebrovascular and neurodegenerative diseases [8,9,10]. Moreover, dietary patterns and food choices typically associated with a Westernised diet, a diet high in fat, sugar, processes foods and refined grains have also been associated with cognitive decline [11], vascular dementia [12,13], Alzheimer’s disease [14], and impaired vascular function. However, to date, there has been limited investigation into the effects of using simple desk-based interruptions to moderate the combined deleterious effects of diet and prolonged sitting on cerebrovascular and cognitive function. 

Recently, our research group assessed the effect of 180-min of sitting with a high and low glycaemic meal on executive function, brain blood flow and cerebral perfusion [3]. Whilst executive function and brain blood flow decreased over time, there was no condition effect suggesting glycaemic index alone did not play a role. However, consumption of a Westernized meal high in refined sugars and triglycerides followed by 180 min of uninterrupted sitting, has been shown to significantly worsen arterial stiffness [15], and independent of sitting, impair cerebrovascular, and executive function [16]. What is not known is whether a simple desk-based mitigation strategy such as leg fidgeting may be of use in offsetting any cerebrovascular and cognitive impairment following a prolonged sitting after consuming a Westernized meal. 

Interrupting prolonged sitting with short and frequent bouts of activity has previously been shown to prevent impairments in vascular health in trials which did not control for meal type [17,18]. For example, leg fidgeting for 1 min on/4 min off over a 180 min period significantly improved endothelial function [19]. In the current study, we propose to determine whether a simple desk-based interruption strategy, leg fidgeting (intervention [INT]), can off-set the detrimental effects on executive and cerebrovascular function posed by prolonged sitting (control [CON]) following a typical Western meal. 

## 2. Materials and Methods

This study is reported in accordance with Consolidated Standards of Reporting Trials guidelines [20]. Institutional ethical approval which adhered to the standards of the journal as well as the Declaration of Helsinki [21] was granted. Written informed consent was provided prior to participating in the study.

### 2.1. Participants

This study recruited a group of healthy young males to minimize any confounding influence of cardiovascular and/or cerebrovascular abnormalities. Participants were excluded if they had any food intolerances, were suffering from any known cardiovascular, metabolic, or cerebrovascular diseases, or were taking any known vascular-acting medications including dietary supplements. As such, 13 young (age = 21.4 ± 1.7 years; BMI = 23.9 ± 2.5 kg/m^2^), physically active (exercising 3.1 ± 1.8 times per week for 2.0 ± 1.2 h per session) healthy males were recruited. All participants were right side dominant. All data was collected between January 2018 and April 2019. 

### 2.2. Experimental Protocol

Each participant visited the laboratory on three separate occasions. During the first visit participants were familiarised with all equipment and experimental procedures including multiple practise trials of the cognitive performance test—the Trail Maker Test (TMT). This was followed by an assessment of height, body mass, and physical activity status, as well as determining any food allergies or intolerances. The following two experimental trials consisting of a Westernised meal, combined with either prolonged uninterrupted sitting (CON) or prolonged sitting with fidgeting (INT) were randomized (using https://www.randomizer.org/, accessed 18 January 2018) and separated by at least 48 h, but no more than 10 days. Participants and assessors were blinded to the condition until the start of their first trial. Each trial began at 08:30 following an overnight fast, consuming only water and having refrained from caffeine for 12 h. Both alcohol and strenuous exercise were abstained for a 24 h period prior to each trial. Prior to the first trial, the evening meal was recorded, and participants were asked to repeat this before their subsequent trial.

For each experimental trial, the participant was asked to empty their bladder and bowel prior to quietly laying on a test bed in a supine position for 20 min. During this time participants were fitted with a continuous wave near infrared spectroscopy (NIRS) (Artinis Medical Systems, BV Zetten, The Netherlands) device over the muscle belly of the dominant gastrocnemius, and the cerebral location FP1 to continuously determine changes in pooling and cerebral perfusion (total haemoglobin (tHb) and de-oxyhaemoglobin (HHb)), respectively. 

In accordance with procedures recommended by Stoner, Barone Gibbs, et al. [22], following the 20 min supine rest, a Doppler ultrasound (Terrason T3300, Burlington, MA, USA) fitted with a linear array probe (15–4 mHz) was used to collect 3 × 10 s videos of the common carotid artery (CCA) on the left side of the body. After this, the participant was manually moved into a seated position using an electronic three-way tilt table (Plinth 2000, Plinth Medical, Suffolk, UK). A baseline blood sample for determination of triglyceride and glucose concentrations were taken from a fingertip. After being seated for a standardized 60 s, participants were asked to complete the Trail Maker Test on an iPad. The participant was then given 10 min to eat their breakfast meal. During the CON trial, participants were asked not to move their lower limbs during the 180-min of sitting. During the INT trial, the participants were asked to fidget their legs to the beat of a metronome at ~250 taps per min, for 1 min on/4 min off, over the entire 180 min sitting period as this was previously shown to improve blood flow and shear rate [19]. A member of the research team informed the participant when to start and stop the 1 min of leg fidgeting throughout the 180 min sitting period. If a participant needed to urinate during the testing protocol, a clinical urine bottle was available. No participants needed to empty their bowels during any testing sessions. Further blood samples for determination of triglyceride concentration were taken at post 170 min post sitting, and glucose was taken at post 30 min, 60 min, 120 min and 170 min of sitting. Following 180 min of sitting participants were lowered back to a supine position for a 10 min period of quiet rest. Following this, all post-assessments were repeated in the same order as the baseline measures. 

## 3. Experimental Procedures

### 3.1. Meal Type

As a traditional Western meal high in refined sugars and fats, all participants consumed a McDonald’s Corporation breakfast meal which included a double sausage and egg McMuffin, two hash browns and a hot chocolate with added double cream (1066 kcal, 4.5 MJ, 61 g fat [of which 20 g was saturated fat], 86 g carbohydrates, 40 g protein and 5 g salt) as this has previously been shown to impair arterial function [15,23]. 

### 3.2. Executive Function

The prefrontal cortex was assessed as it plays an important role in cognitive function and executive functions [24]. Executive functions are a key facet of cognition, and are key for everyday situations that require complex organisational skills including problem solving, maintaining attention, and task switching [25]. Executive functions have been shown to be improved with acute bouts of physical activity [26]. Cognitive processing and executive function were assessed in the current study using the TMT. The TMT is split into two parts; Part-A (associated with cognitive processing) and Part-B (associated with executive function) [27]. Additionally, the difference in completion time (s) between TMT A and B reflects the additional cognitive control required to switch between sequential numbers and letters and is considered executive control [28]. A reduction in TMT B relative to A has been suggested to indicate cognitive impairment [29]. TMT A presents 25 encircled numbers 1–25, the participant is required to tap each one in numerical order. For TMT B, numbers and letters are presented in a semi-randomized order on the screen. The participant had to connect 25 encircled numbers and letters in numerical and alphabetical order, alternating between the numbers and letters. These tests were practised during the familiarisation session and then for the experimental trials they were conducted once pre (0-min) and post (180-min) sitting. For each TMT A and B, time to completion, number of errors, and mental fatigue were measured. Acceptable reliability (ICC: 0.85) for these measures has been previously reported in healthy adults [30]. 

### 3.3. Common Carotid Arterial Blood Flow 

A trained ultrasound operator with extensive experience (>15 years) collected all CCA measurements using ultrasound. Three 10 s videos of the ultrasound readings were recorded using external video capturing software (LiteCam HD, Englewood Cliffs, NJ, USA). The video clips were analyzed offline using automated edge-detecting software (FMD Studio, Quipu, Italy) by a trained operator blinded to the condition and time point. The operator had 15 years’ experience. Custom written Excel Visual Basic code was used to fit peaks and troughs to the diameter waveforms in order to calculate diastolic, systolic, and mean diameters. Blood flow was calculated from continuous diameter and mean blood velocity recordings using the equation: 3.14 × (diameter/2)^2^ × mean blood velocity × 60. 

### 3.4. Near Infrared Spectroscopy (NIRS)

A trained operator with extensive experience (>15 years) collected all NIRS measurements. In order to determine the tHb concentration, which was used to indicate changes in perfusion in the head (FP1) and leg (gastrocnemius), a continuous wave NIRS device using spatially resolves spectroscopy was employed (Artinis, Portalite, Einsteinweg, The Netherlands). In spatially resolved spectroscopy the spatial profile of the intensity of backscattered light is determined as a function of the distance from the light transmitter, the shape of this function being related to the absorption coefficient, from which tHb can be determined [31]. Changes in tHb and HHb using NIRS have previously been shown to be both valid and reliable during an orthostatic challenge [31]. It should be noted that as NIRS cannot distinguish between O_2_Hb and oxy-myoglobin; for clarity this paper will refer to the combination of both as Hb. 

Previously, studies have reported either tHb or HHb as a measure of perfusion [32,33]; so for comparison reasons, the current study reports both. The gastrocnemius optode was placed in the vertical plane over the central belly of the muscle at the position of maximum circumference in accordance with Stone, Fryer, Ryan, Stoner [31]. Depending on head geometry, the FP1 optode was placed over the right eyebrow in accordance with the 10–20 system of electrode placement [34]. To ensure the same optode location was used between visits, photos were used in conjunction with the anatomical measurements. Cerebral and leg perfusion were assessed pre and post sitting, and AUC was calculated as a global measure during each sitting protocol. As a measure of cerebral responsiveness to the executive function tests, the change in perfusion which occurred during the TMT Part A and B was calculated as pre-TMT tHb-average-TMT tHb, and this is reported as Δ head tHb; the same was conducted for HHb. 

### 3.5. Blood Sampling

After a 1.6 mm blood lancet (Haemedic, Poland) punctured the fingertip, samples for determination of triglyceride concentration (mg·dL) were collected from the capillary using a 32 μL lithium heparin capiliette (Sarstedt Aktiengesellschaft & Co., Nümbrecht, Germany). All samples were immediately extracted onto a Reflotron test strip (Hoffmann-La Roche Ltd., Basel, Switzerland) for analysis using a Reflotron Plus (Hoffmann-La Roche Ltd.). Using the same sample site, glucose concentration (mmol·L^−1^) was determine by collecting 20 μL of blood in a lithium heparin capiliette (Sarstedt Aktiengesellschaft & Co., Nümbrecht, Germany), this was immediately analysed using a Biosen C-Line device (EKF Diagnostics, Cardiff, UK). 

### 3.6. Sample Size

Using a previously reported effect size of 0.4 derived from our previous study assessing executive function [3], and power set at 0.8 with Type I error set at 0.05, approximately 12 participants were required. We over-recruited by n = 1 to account for any data errors or potential attrition during the study. 

### 3.7. Statistical Analysis

Statistical analyses were performed using Statistical Package for Social Sciences version 25 (SPSS, Inc., Chicago, IL, USA). The investigating team were blinded to each condition (CON vs. INT) until all statistical analysis were completed. Using the Shapiro–Wilk and Mauchly’s test of sphericity, all dependent variables were found to be normally distributed and spherical. Data are reported as mean, standard deviation (SD), mean difference (MD) and 95% confidence intervals (CI) unless otherwise stated. For all two-way repeated measures ANOVAs where a significant interaction of time x condition was found, two separate one-way ANOVAs with post-hoc Bonferroni were conducted on each condition. When a significant time or condition effect was reported, paired samples *t*-tests with MD and 95% CI were reported. As measures of effect size for ANOVA, partial Eta^2^ (η_p_^2^) was used where 0.01, 0.06, and 0.14 represent a small, medium, and large effect, respectively [35]. As measures of effect size for *t*-tests, Cohens’*d* was used where 0.2, 0.5 and 0.8 represent a small, medium, and large effect, respectively [35]. The α was set at *p* < 0.05 (two tailed).

## 4. Results

### 4.1. Triglyceride and Glucose Concentration

As shown in Table 1 there was a non-significant interaction for triglyceride concentration (η_p_^2^ = 0.206, *p* = 0.077). There was also a significant main effect of time (η_p_^2^ = 0.630, *p* = 0.0001; MD = 51.4, 95% CI = 28.8–74 mg·dL^−1^), but not condition, with triglyceride concentration increasing from pre to post in both the CON (pre = 73 ± 5; post = 137 ± 56 mg·dL^−1^) and INT (pre = 73 ± 9; post = 112 ± 50 mg·dL^−1^) trials.

There was a non-significant interaction (η_p_^2^ = 0.047, *p* = 0.703) and condition (η_p_^2^ = 0.019, *p* = 0.652) effects for glucose. There was a significant time effect (η_p_^2^ = 0.715, *p* < 0.0001) with post-hoc analyses finding a spike at post 30 which was significantly higher than pre (*p* = 0.0001; MD = 1.67, 95% CI = 0.88–2.45 mmol·L^−1^), post 60 (*p* < 0.0001; MD = 1.079, 95% CI = 0.559–1.598 mmol·L^−1^), post 120 (*p* = 0.001; MD = 1.27, 95% CI = 0.553–1.987 mmol·L^−1^) and post 170 min (*p* < 0.0001; MD = 1.823, 95% CI = 0.913–2.734 mmol·L^−1^). 

### 4.2. Measures of Cognitive Functions

#### 4.2.1. Trail Maker Part-A

For the dependent variable, completion time, (s) there was a non-significant interaction of condition x time, and main effect of time, but a significant effect of condition. For the dependent variables number of errors, fatigue score and Δ head tHb, there were no significant interaction or main effects.

#### 4.2.2. Trail Maker Part-B

As shown in Table 2, there was a significant interaction of condition x time for completion time. Follow-up analyses found completion time significantly increased over 180 min of sitting in the CON trial (MD = 5.2, 95% CI = 1.6–8.7 s, d = 0.38) and significantly decreased over 180 min for the INT trial (MD = 2.3, 95% CI = −1.7–6.2 s, d = 0.97). For the dependent variable number of errors there was a significant and meaningful interaction of condition x time. Follow-up analyses found the number of errors significantly increased over time in the CON trial (MD = 3.33, 95% CI = 0.25–6.41, d = 0.68) with no significant change over time in the INT trial. For the dependent variable fatigue there was a significant and meaningful interaction of condition x time. Follow-up analyses found that fatigue significantly increased over time in the CON trial (MD = 0.73, 95% CI = 0.20–1.26, d = 0.92) with no change over time for the INT trial. For the dependent variable TMT B-A, there were no significant interactions or main effects.

### 4.3. Carotid Artery and Cerebral Perfusion Measures

As presented in Table 3, there were no significant or meaningful interactions or main effects for the dependent variables carotid blood flow, diameter and velocity, or cerebral perfusion determined as tHb and HHb. 

### 4.4. Blood Pooling

As presented in Table 4 there was a significant difference between the CON and INT trials for both AUC tHb (MD = 61.30, 95% CI = 31.72–90.88 µmmol·L^1^), and AUC HHb (MD = 44.75, 95% CI = 12.49–77.01 µmmol·L^1^) assessed at the leg. There were no significant differences in AUC for tHb or HHb assessed at the head.

## 5. Discussion

The aim of the current study was to determine whether a simple desk-based interruption strategy, leg fidgeting (INT), can off-set the detrimental effects on executive and cerebrovascular function posed by prolonged uninterrupted sitting following a typical Western meal (CON). As expected, for the CON trial, consuming a Western meal high in refined sugars and fat resulted in elevated postprandial glucose and triglyceride concentrations during uninterrupted prolonged sitting. The main finding of the current study is that following consumption of a Western meal, leg fidgeting (INT) during prolonged sitting significantly improved, or maintained executive functions compared to CON as assessed by the TMT Part B. Processing speed (TMT Part A) was not affected by the CON or INT trials. There were no significant pre-post changes in cerebral perfusion in either the CON or INT trials. However, the change in cerebral perfusion which occurred during the TMT B (Δ tHb) was smaller after 180-min sitting compared to the start, with no difference between trials. Leg fidgeting significantly reduced pooling in the leg (tHb and HHb), but did not significantly alter the glucose or triglyceride responses. There were no significant changes in CCA blood flow. 

### 5.1. Limitations and Strengths

Whilst this is the first study to a to assess whether regular leg fidgeting can off-set the detrimental effects on executive and cerebrovascular function posed by prolonged sitting following a typical Western meal, in order to better contextualise the discussion, it is important to first state the known limitations and strengths of the study. First, as the population are all habitually active males, findings cannot be generalised beyond this group. Second, our study used a limited number of cognitive tests rather than an extensive battery and as such we may have missed a component of executive function. However, we have still expanded and built upon the existing research in the area, and we likely avoided questionnaire fatigue which can be common with extensive test batteries [36]. Third, unlike previous research assessing executive function with prolonged sitting, our study accounted for the proposed learning effect [37] caused by not using familiarisation sessions. Lastly, a significant strength of this study was that we employed leg fidgeting as an intervention which is a practical, low-cost and easy to adopt rather than walking, standing or simple resistance activities that either require time away from work or monetary investment. 

### 5.2. Comparison to the Literature

The current study found that after consumption of a Western meal, leg fidgeting was able to significantly improve a number of measures of executive function. Specifically, Table 2 shows that during the CON trial, relative completion time was slower by 34%, the number of errors increased by 88% and participants felt 21% more fatigued. However, during the INT trial, leg fidgeting for 1 min on/4 min off improved completion time by 13%, decreased the number of errors by 47% and participants felt 6% less fatigued compared to baseline. Our results build upon those of Wanders, Cuijpers, et al. [38] who found that after 4-h of sitting with different Western meal types, physical activity interruption delayed post-breakfast mood and vigour decrements, as well as increases in fatigue and sleepiness irrespective of meal type. However, our findings are also in direct conflict to others [3] who assessed the effects of a high and low glycaemic beverage on TMT B pre and post 180 min of uninterrupted sitting. The authors found that irrespective of the beverage type, completion time significantly improved over 180 min of sitting. The differences in findings between this study and ours may be explained by the dissimilar protocols used. Burnet, Blackwell, et al. [3] did not report TMT familiarisation prior to their experimental trials, whereas in the current study there was prior practise during the familiarization visit in order to try and remove any learning effect. Learning effects with the TMT have previously been reported. Dye [37] observed that in a healthy population, improvements in both parts A and B were found when the participants repeated the test twice on the same day. As such, the participants used by Burnet, Blackwell, et al. [3] may have experienced a learning effect which could in-part explain the faster completion time over the 180 min [39]. Differences may also be due to the composition of the meals, given that Burnet, Blackwell, et al. [3] compared a low and high glycaemic beverage, and our Western meal has both a high glycaemic index and a high-fat content, the combination of the two may cause an exacerbated response. Previously, it was reported that Western meals high in both fats and refined sugars impair several aspects of cognition including working memory, attention and inhibitory control, as well as contributing to neurodegenerative conditions [16].

As there were no pre vs. post changes in cerebral perfusion (tHb & HHb) or CCA blood flow, velocity or diameter, these variables are unlikely to independently explain the significant changes in executive function assessed using the TMT B in the current study. However, as shown in Table 2, the change (Δ head tHb) in cerebral perfusion that occurred during the TMT Part B (determined as pre tHb–average tHb), was significantly reduced over time irrespective of condition. It may be that the impairment in executive function caused by the CON trial, and its subsequent improvement with leg fidgeting is not as heavily influenced by hemodynamic changes as initially thought. Though the importance of this exploratory cerebral measure is currently not known. One possible explanation maybe related to leg fidgeting being such a low intensity of physical activity, and therefore not being a great enough stimulus to sufficiently increase systemic blood flow and oxygenation. Previous research has reported that higher intensity exercise (moderate-vigorous intensity) improved both cognition and task-efficient oxygenation in the prefrontal cortex [40,41]. Another explanation why there was no change in Δ head tHb between conditions, may be related to increased feelings of sleepiness, mental effort and mental fatigue which have previously been reported to increase with prolonged sitting [42]. However, the authors found that standing breaks did not alleviate these sitting induced impairments. It may be that, again, more vigorous/frequent exercise is needed to off-set sleepiness, mental effort and fatigue. In the current study, rapid and frequent leg fidgeting was found to offset the detriment in fatigue (Table 2) seen during the CON trial. However, as we did not assess sleepiness or mental effort, we cannot speculate about the potential wider benefits of leg fidgeting. As such, further research is warranted to determine the psychological and physiological mechanisms that may influence feelings of sleepiness, mental fatigue and effort caused by sitting with and without interruption.

## 6. Conclusions

Previous studies have associated chronic sedentary behaviours with increased dementia risks [43] and cognitive impairment [2], but less is known about the influence that modifiable lifestyle behaviours may have on these functions [3]. Specifically, there is limited knowledge about how simple physical activity strategies such as leg fidgeting may help to alleviate compounded dysfunctions caused by prolonged sitting and Western meals high in refined sugars and fats. As expected, findings from the current study suggest that 180 min of uninterrupted sitting following a Western meal which is high in saturated fats and refined sugars impairs executive functions, and increases feelings of fatigue. These significant impairments are off-set, or improved when sitting is interrupted with leg fidgeting for one minute of every five. As such, leg fidgeting may be a viable desk-based option for maintaining and improving executive function for office workers who chose to consume in meals high in fats and refined sugars prior to prolonged periods of sitting.

## Figures and Tables

**Table 1:** Mean (SD) glucose concentrations during the control and intervention trials.

	Mean (SD) Glucose Concentration (mmol·L^−1^)				
	Pre *	Post 30 min	Post 60 min *	Post 120 min *	Post 170 min *
**CON**	4.54 (0.38)	6.08 (0.87)	5.04 (0.88)	4.81 (0.85)	4.49 (0.50)
**INT**	4.39 (0.49)	6.18 (0.79)	5.06 (0.67)	4.91 (0.67)	4.13 (0.91)

* = combined means are significantly (*p* < 0.05) different from post-30 min; CON = control trial; INT = intervention trial.

**Table 2:** Mean (SD) measures of cognitive function (Trail Maker Part-A and Part-B), and change (Δ) in cerebral perfusion pre and post the control and intervention trials.

	Trail Maker Part-A					Trail Maker Part-B					TMT B-A
	Completion Time (s)	Number of Errors	Fatigue Score	Δ Head tHb (µmol·L^−1^)	Δ Head HHb (µmol·L^−1^)	Completion Time (s)	Number of Errors	Fatigue Score	Δ Head tHb (µmol·L^−1^)	Δ Head HHb (µmol·L^−1^)	TMT B-A (s)
Mean Average (SD)											
**CON**											
0 min	11.99 (2.05)	0.9 (0.99)	2.7 (1.25)	2.13 (1.35)	−0.03 (0.58)	15.97 (3.86)	0.42 (0.51)	2.64 (1.21)	2.66 (1.17)	−0.36 (0.60)	3.97 (4.02)
180 min	12.81 (2.72)	2.2 (3.05)	3.3 (1.42)	1.33 (2.20)	−0.02 (0.81)	21.14 (4.95)	3.75 (4.81)	3.36 (1.63)	1.28 (1.63)	−0.29 (0.97)	8.32 (4.52)
**INT**											
0 min	11.17 (1.23)	0.3 (0.67)	2.9 (1.29)	1.65 (1.94)	−0.09 (0.44)	17.52 (6.29)	1.58 (3.20)	3.00 (1.26)	2.10 (1.89)	−0.37 (0.62)	6.36 (6.41)
180 min	10.31 (0.84)	0.6 (1.27)	2.8 (1.48)	1.49 (2.60)	0.46 (0.58)	15.27 (3.61)	0.83 (1.34)	2.81 (1.40)	0.41 (1.95)	−0.01 (0.57)	4.96 (3.53)
**Interaction Effect (Time × condition)**											
*p*	0.067	0.430	0.111	0.605	0.090	0.021 *	0.042 *	0.049 *	0.601	0.192	0.059
η_p_^2^	0.297	0.070	0.258	0.035	0.318	0.492	0.324	0.325	0.036	0.202	0.312
**Time Effect**											
*p*	0.976	0.207	0.213	0.487	0.124	0.175	0.084	0.140	0.047 *	0.291	0.148
η_p_^2^	<0.001	0.171	0.167	0.062	0.185	0.176	0.246	0.205	0.409	0.138	0.197
**Condition Effect**											
*p*	0.049 *	0.068	0.394	0.784	0.088	0.141	0.369	0.588	0.232	0.491	0.647
η_p_^2^	0.335	0.323	0.082	0.010	0.320	0.204	0.074	0030	0.173	0.061	0.022

SD = standard deviation; η_p_^2^ = partial Eta^2^; *p* = significance; * = *p* < 0.05; tHb = total haemoglobin; HHb = deoxygenated haemoglobin; Δ head tHb = the difference in tHb between immediately pre and the average during the trail maker test; CON = control trial; INT = intervention trial.

**Table 3:** Mean (SD), interaction of time × condition, and main effect of time for carotid ultrasound measures and cerebral perfusion pre (0 min) and post (180 min) the control and intervention trials.

	Carotid Blood Flow (mL·min^−1^)	Carotid Avg. Diameter (mm)	Carotid Velocity (m·s^−1^)	Cerebral Perfusion tHb (µmol·L^−1^)	Cerebral Perfusion HHb (µmol·L^−1^)
**Mean Average (SD)**					
**CON**					
0 min	677 (182)	6.29 (0.27)	39.82 (7.75)	1.55 (1.87)	0.44 (0.84)
180 min	613 (157)	6.29 (0.33)	35.1 (5.59)	−0.05 (8.99)	1.80 (2.91)
**INT**					
0 min	641 (186)	6.36 (0.44)	37.17 (9.3)	1.74 (1.54)	1.11 (3.14)
180 min	621 (143)	6.32 (0.29)	32.93 (14.67)	−2.06 (5.89)	1.40 (4.68)
**Interaction Effect (Time × Condition)**					
*p*	0.335	0.718	0.926	0.576	0.476
η_p_^2^	0.116	0.014	0.001	0.041	0.065
**Time Effect**					
*p*	0.321	0.724	0.240	0.108	0.124
η_p_^2^	0.123	0.013	0.149	0.290	0.269
**Condition Effect**					
*p*	0.687	0.527	0.226	0.662	0.920
η_p_^2^	0.021	0.041	0.135	0.025	0.001

SD = standard deviation; η_p_^2^ = partial Eta^2^; *p* = significance; CON = control trial; INT = intervention trial.

**Table 4:** Mean (SD) area under the curve tHb and HHb in the head and leg during the control and intervention trials.

	CON Mean Average (SD)	INT Mean Average (SD)	*p*	Cohens’*d*
**Leg AUC tHb (** **µmol** **·L^1^)**	261.5 (84.7)	200.2 (31.1)	0.001 *	1.392
**Leg AUC HHb (** **µmol** **·L^1^)**	207.9 (78.5)	163.2 (72.5)	0.011 *	0.881
**Head AUC tHb (** **µmol** **·L^1^)**	−20.1 (74.2)	7.2 (95.3)	0.540	0.213
**Head AUC HHb (** **µmol** **·L^1^)**	28.4 (29.5)	18.6 (54.1)	0.626	0.160

AUC = area under the curve; SD = standard deviation; *p* = significance; * = *p* < 0.05; CON = control trial; INT = intervention trial.

## Data Availability

Data is available upon request to the corresponding author.
